# Anti-phospholipids antibodies and immune complexes in COVID-19 patients: a putative role in disease course for anti-annexin-V antibodies

**DOI:** 10.1007/s10067-021-05580-3

**Published:** 2021-01-19

**Authors:** Antonio Cristiano, Valentina Fortunati, Fabio Cherubini, Sergio Bernardini, Marzia Nuccetelli

**Affiliations:** 1 Department of Experimental Medicine, Tor Vergata University, Via Montpellier, 1, 00133 Rome, Italy; 2 Department of Biomedicine and Prevention, Tor Vergata University, Rome, Italy; 3 Tor Vergata University Hospital, Rome, Italy; 4 Emerging Technologies Division, International Federation of Clinical Chemistry and Laboratory Medicine (IFCC), Milano, Italy

**Keywords:** Anti-phospholipid antibodies, Coagulopathy, COVID-19, Thrombotic events

## Abstract

**Introduction:**

Besides distinctive respiratory and digestive hallmarks, COVID-19 has been recently associated with a high prevalence of pro-inflammatory and hypercoagulable states known as “COVID-19 Associated Coagulopathy” (CAC), corresponding to a worsening in patients’ conditions, whose causes are still to be elucidated. A link between anti-phospholipid antibodies (aPLs) and viral infections has long been suggested. APLs are assessed for anti-phospholipid syndrome (APS) diagnosis, characterized by thrombocytopenia, thrombosis, and coagulopathy. Furthermore, circulating immune complexes (CICs), arisen upon inflammatory responses and related immune dysregulation, can lead to endothelial cell damage and thrombotic complications.

**Method:**

We performed an extended panel including IgG/IgM anti-cardiolipin, IgG/IgM anti-β2-glycoprotein-1, coupled with IgG/IgM anti-prothrombin, IgG/IgM anti-annexin-V on two COVID-19 patient groups (early and late infection time), and a negative control group. IgG CIC analysis followed to evaluate inflammatory status, through a possible complement system activation.

**Results:**

Our results showed low positive case percentage in IgG/IgM anti-cardiolipin and IgG/IgM anti-β2-glycoprotein-1 assays (4.54%, 6.25%, and 4.55%; in early infection group, late infection group, and control group, respectively); few positive cases in IgG/IgM anti-prothrombin and IgG/IgM anti-annexin-V immunoassays; and no IgG CIC positivity in any patient.

**Conclusions:**

In conclusion, our data show a low aPL prevalence, likely excluding an involvement in the pathogenesis of CAC. Interestingly, IgG/IgM anti-prothrombin and anti-annexin-V positive cases, detected in late infection group, suggest that aPLs could temporarily increase or could trigger a “COVID-19-induced-APS-like-syndrome” in predisposed patients.

**Table:** 

**Key Points** **•** *To our knowledge, anti-prothrombin (aPT) antibodies, anti-annexin-V antibodies and CICs in COVID-19 patients have not been reported in the scientific literature.* **•** *Lack of uniformity and the low percentage of aCL/aβ2GP1 positivity preclude a putative role in CAC pathogenesis.* **•** *IgG/IgM anti-prothrombin and IgG/IgM anti-annexin-V data show that distribution of positive case number increases in late infection patients, significantly in anti-annexin-V results, suggesting a possible role for these anti-phospholipid antibodies in disease course.* **•** *aPLs can arise transiently in some patients with critical illness and SARS-CoV-2 infection (disappearing in a few weeks), as well as in other genetically predisposed patients; they could trigger a “COVID-19-induced-APS-like-syndrome”.*

## Introduction

Severe Acute Respiratory Syndrome Coronavirus 2 (SARS-CoV-2) is a new β-coronavirus strain identified for the first time in Wuhan, China, on late December 2019. On February 2020, the World Health Organization (WHO) named the infectious disease “coronavirus disease 2019” (COVID-19) [1].

In a second time, a high prevalence of coagulation abnormalities and thrombotic complications has been found in COVID-19. These clinical manifestations have been called “COVID-19 Associated Coagulopathy” (CAC), corresponding to a general worsening of patient conditions, that might increase the risk of deep vein thrombosis (DVT), pulmonary embolism (PE), and disseminated intravascular coagulation (DIC) [2, 3]. Indeed, presence of small- and mid-sized pulmonary artery thrombosis and microangiopathy was found in COVID-19 patients, due to markedly low levels of oxygen. Subsequent respiratory failure causes Intensive Care Unit (ICU) admission and the need for mechanical ventilation [4–6].

CAC is characterized by increased levels of routine clinical inflammation markers such as C reactive protein (CRP), erythrocyte sedimentation rate (ESR), together with acute inflammatory response (cytokine storm): interleukin-6 (IL-6), interleukin-1 (IL-1), and tumor necrosis factor α (TNFα). IL-6 levels are significantly higher in patients with severe conditions [7, 8].

Regarding clinical laboratory data, D-dimer and fibrinogen levels are frequently elevated in these patients, and other coagulation parameter abnormalities, such as activated partial thromboplastin time (aPTT), antithrombin (AT), and prothrombin time (PT), have also been described in critically ill patients [7–11].

We can hypothesize three different pathological ways in CAC pathogenesis: (i) SARS-CoV-2 infection in endothelial cells may cause loss of endothelial homeostasis and its physiological anticoagulant activity; (ii) the systemic inflammatory response and cytokine storm may increase atherosclerotic plaque rupture probability, in patients with previous cardiovascular events history; (iii) anti-phospholipid antibodies (aPLs) along with the development of circulating immune complexes (CICs) could be involved in thrombosis events.

We focused our study on the last hypothesis.

Anti-phospholipid antibody positivity clinical manifestations include thrombosis, thrombocytopenia, coagulopathy, and pregnancy complications with recurrent spontaneous abortions.

Furthermore, anti-phospholipid antibodies are essential clinical criteria in the anti-phospholipid syndrome (APS) diagnosis, a systemic autoimmune disease in which specific laboratory markers, such as lupus anticoagulant (LAC), IgG and/or IgM anti-cardiolipin (aCL), and IgG and/or IgM anti-β2-glycoprotein-1 (aβ2GP1) antibodies, are crucial to the diagnosis. These antibodies in fact represent the most frequent aPLs. IgG/IgM anti-prothrombin (aPT) and IgG/IgM anti-annexin-V are also detected in a minority of cases, especially in clinical APS patients with negative classical anti-phospholipid antibodies. In healthy population, positivity to anti-phospholipid antibodies has been found in about 5% of cases, with an uncertain relationship for increased risk to develop thrombotic events and APS. Complement activation is also required for the full APS clinical manifestation.

In addition, inflammatory responses and related immune dysregulation could trigger the development of circulating immune complexes (CICs), which leads to endothelial cell damage and organ inflammation through their tissue depositing and through complement system activation (C1q, C3), resulting in thrombotic complications. Moreover, macrophages could phagocytose CICs causing a hyperinflammatory response, typical in COVID-19 patients [12].

To our knowledge, anti-prothrombin (aPT), anti-annexin-V antibodies, and CICs in COVID-19 patients have not been reported in the scientific literature.

In this perspective, to better assess the anti-phospholipid antibodies’ role in CAC, we performed a more extended study panel including classical aPLs (IgG/IgM anti-cardiolipin and IgG/IgM anti-β2-glycoprotein-1), supported by other anti-phospholipid antibodies (IgG/IgM anti-prothrombin and IgG/IgM anti-annexin-V). We also detected IgG human circulating immune complexes (CICs) to evaluate the inflammatory status, through a possible complement system activation. These antibodies were assessed on a COVID-19 patients’ cohort compared with a control group (healthcare workers).

## Patients and methods

Serum samples were recovered in accordance with local ethical approvals (R.S.44.20), from “Tor Vergata” University COVID-Hospital of Rome hospitalized patients as follows: 44 positive RT-PCR-diagnosed SARS-CoV-2 patients, collected on days 1 to 9 from first access to Emergency Department and from first positive nasopharyngeal swab (early infection patients); 48 positive RT-PCR-diagnosed SARS-CoV-2 patients, collected on days 19 to 41 from first access to Emergency Department and from first positive nasopharyngeal swab (late infection patients); and 44 negative RT-PCR-diagnosed SARS-CoV-2 subjects (control group) collected from “Tor Vergata” Hospital physicians and healthcare workers screened for internal surveillance.

All samples were randomly selected and were collected from March 16, 2020 to April 28, 2020.

All enrolled patients were hospitalized, and in particular, 17/44 in early infection group and 13/48 in late infection group were admitted to respiratory system department; none required ICU admission and/or mechanical ventilation.

All subjects of the groups had been tested by serological assays for IgM and IgG anti-SARS-CoV-2 antibody detection (submitted data). Early and late infection groups are composed by different individuals.

Informed consent was obtained from all subjects enrolled in the study. Sera were separated by centrifugation at 2500*g* for 10 min, within 1 h from collection. The study was in accordance with the Helsinki Declaration, as revised in 2013.

### Chemiluminescence immunoassay

Semi-quantitative determination of IgG/IgM aCL and IgG/IgM aβ2GP1 antibodies in human serum was performed on the fully automated BIO-FLASH® instrument (Inova Diagnostics, San Diego, USA) with QUANTA Flash® APS-aCL family and aβ2GP1 family reagents (Inova Diagnostics, San Diego, USA). The QUANTA Flash aCL and aβ2GP1 assays are chemiluminescent two-step immunoassays consisting of magnetic particles coated with cardiolipin and human-purified β2GP1 proteins which capture, if present, aCL and aβ2GP1 anti-phospholipid antibodies from the sample. The emitted light is measured as relative light units (RLUs) by the BIO-FLASH optical system; RLUs are directly proportional to the aCL and aβ2GP1 concentration in samples: manufacturer’s recommended cut-off > 20 CU (chemiluminescent units).

### Enzyme-linked immunosorbent assay

Immunoenzymatic assay “Prothrombin IgG/IgM ELISA kit” (Demeditec Diagnostics GmbH, Kiel, Germany) was performed for quantitative measurement of IgG and IgM autoantibodies against prothrombin proteins in human serum. Standards and diluted samples (1:100) were incubated for 30 min in wells coated with prothrombin antigens, allowing the binding to the specific IgG/IgM prothrombin antibodies. After washing, a conjugate solution labeled with horseradish peroxidase (HRP) was dispensed into each well for 15 min. Finally, a chromogenic solution containing HRP substrate (tetramethylbenzidine; TMB) was added for 15 min, and the reaction was then stopped by an acidic solution. The absorbances were read spectrophotometrically at 450 nm on a Plate Reader (DAS srl, Rome, Italy). Optical densities are proportional to the quantity of specific IgG/IgM prothrombin antibodies present in the samples. The results were estimated from a calibration curve (0, 6.3, 12.5, 25, 50, 100 U/ml). IgG and IgM anti-prothrombin manufacturer’s recommended cut-off values were > 12 U/ml.

Immunoenzymatic assay “Eu-Annexin G/M” (Eurospital, Trieste, Italy) was performed for quantitative measurement of IgG and IgM antibodies against annexin-V in human serum. Standards and prediluted samples (1:100) were pipetted into wells precoated with purified annexin-V. After 30-min incubation at room temperature, the microwell contents were discarded and a conjugate solution labeled with horseradish peroxidase was dispensed for 15 min. At the end of incubation, TMB was added for 15 min and the reaction was then stopped by an acidic solution. The absorbances of the colorimetric reaction were read at 450 nm on a Plate Reader (DAS srl, Rome, Italy). Optical densities are proportional to the quantity of specific IgG/IgM annexin-V antibodies present in the samples. The results were calculated on the corresponding standard curve (0, 6.3, 12.5, 25, 50, and 100 U/ml). IgG and IgM anti-annexin-V manufacturer’s recommended cut-off values were > 8 U/ml.

The immunoenzymatic assay “CIC-C1q ELISA (IgG)” (EUROIMMUN, Lubeck, Germany) was performed for quantitative determination of human circulating immune complexes, containing IgG antibodies directed against C1q protein. Standards and diluted samples (1:51) were incubated into microplate wells coated with C1q protein for 30 min. After washing, a conjugate solution labeled with horseradish peroxidase was dispensed into each well for 30 min. Finally, a chromogenic solution containing HRP substrate (tetramethylbenzidine; TMB) was added for 15 min, and the reaction was then stopped by an acidic solution. The absorbances of the colorimetric reaction were read at 450 nm on a Plate Reader (DAS s.r.l., Rome, Italy) within 30 min, and the results were calculated on the corresponding standard curve (2, 20, and 200 RU/ml; RU = relative units). IgG CIC manufacturer’s recommended cut-off value was > 20 RU/ml.

### Statistical analysis

Results were calculated by Mann-Whitney test. More than two-group comparison was determined by non-parametric one-way ANOVA test (Kruskal-Wallis test). Statistical significance was defined as *p* < 0.05. All data were analyzed using GraphPad Prism Software 8.4.3 (San Diego, California, USA). The investigators were blinded to the group allocation during the experiment.

## Results

We analyzed a total of 92 SARS-CoV-2 positive patients and 44 negative controls (both confirmed by RT-PCR). In all groups, we first tested IgG/IgM aCL and IgG/IgM aβ2GP1 prevalence, performing a semi-quantitative automated chemiluminescent assay.

In positive early infection group (*n*=44; 25 males and 19 females; mean age 67.3 years ± 16.6 years), 2 patients (4.54%) were positive to IgG/IgM aCL or IgG/IgM aβ2GP1: one with IgG aCL = 27.9 CU; one with IgM aCL = 34.3 CU and IgM aβ2GP1 = 31.5 CU.

In positive late infection group (*n* = 48; 27 males and 21 females; mean age 69.7 years ± 13.3 years), 3 patients were positive (6.25%) to IgG/IgM aCL or IgG/IgM aβ2GP1. In particular, one had IgG aCL = 39.9 CU, one had IgM aβ2GP1 = 30.1 CU, and one had IgG aCL = 31.9 CU.

In negative control group (*n* = 44; 23 males and 21 females; mean age 41.7 years ± 11.1 years), we detected 2 patients (4.55%) with anti-phospholipid antibody positivity: one patient had IgG aCL = 56.7 CU; one patient had IgM aCL = 41.5 CU and IgM aβ2GP1 = 26.9 CU.

**Fig. 1:**
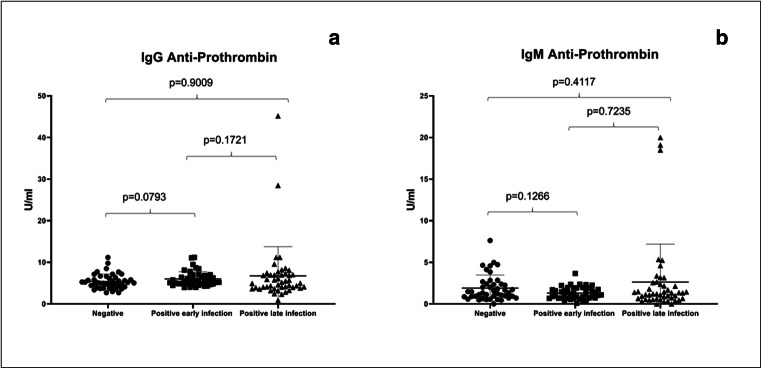
IgG anti-prothrombin results in control group, positive early infection group, and positive late infection group (**a**) (Kruskal-Wallis *p* value = 0.1938); IgM anti-prothrombin results in control group, positive early infection group, and positive late infection group (**b**) (Kruskal-Wallis *p* value = 0.3584)

**Fig. 2:**
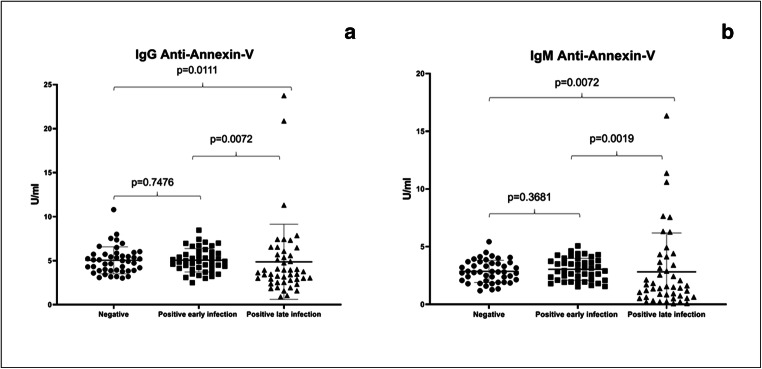
IgG anti-annexin-V results in control group, positive early infection group, and positive late infection group (**a**) (Kruskal-Wallis *p* value = 0.0101); IgM annexin-V results in control group, positive early infection group, and positive late infection group (**b**) (Kruskal-Wallis *p* value = 0.0029)

Next, we assessed IgG/IgM anti-prothrombin and IgG/IgM anti-annexin-V prevalence on the different groups with quantitative ELISA assays. Results are shown in Figs. 1 and 2, respectively.

The IgG and IgM anti-prothrombin median concentrations (Table 1) did not show a statistical significance among the groups (*p* = 0.1938 and *p* = 0.3584, respectively). No patient with IgG/IgM anti-prothrombin antibody positivity was found in negative control group and in positive early infection group. In positive late infection group, two patients (4.16%) showed IgG prothrombin antibody positivity (45.2 U/ml; 28.5 U/ml) and three patients showed IgM prothrombin antibody positivity (19.1 U/ml, 20 U/ml, and 18.5 U/ml) (Fig. 1).

**Table 1:** IgG/IgM anti-prothrombin, IgG/IgM anti-annexin-V, IgG CIC median concentration, and range

	Negative controls (*N* = 44)	Positive early infection patients (*N* = 44)	Positive late infection patients (*N* = 48)
IgG anti-prothrombin	5.110 U/ml (range 2.720–11.20)	5.475 U/ml (range 3.960–11.20)	5.020 U/ml (range 0.920–45.20)
IgM anti-prothrombin	1.405 U/ml (range 0.007–7.630)	1.150 U/ml (range 0.100–3.660)	1.220 U/ml (range 0.007–20.00)
IgG anti-annexin-V	4.967 U/ml (range 3.023–10.80)	4.933 U/ml (range 2.507–8.467)	3.638 U/ml (range 0.900–23.75)
IgM anti-annexin-V	2.812 U/ml (range 1.190–5.433)	2.957 U/ml (range 1.537–5.067)	1.531 U/ml (range 0.075–16.35)
IgG CICs	1.045 RU/ml (range 0.1000–10.30)	0.66 RU/ml (range 0.1000–3.940)	1.120 RU/ml (range 0.2900–9.660)

IgG/IgM anti-annexin-V assay results (Table 1) showed statistically significant median concentrations among the groups (*p* = 0.0101 and *p* = 0.0029, respectively). IgG weak positivity (2.27%) was found in one patient, both in negative control group and early infection group (10.8 U/ml and 8.47 U/ml, respectively). In positive late infection group, three patients (6.25%) showed IgG anti-annexin-V antibody positivity (23.75 U/ml, 11.31 U/ml, and 20.87 U/ml); moreover, three patients showed IgM anti-annexin-V antibody positivity (16.25 U/ml, 11.37 U/ml, and 10.6 U/ml). This group showed statistically significant *p* values when compared separately with control group and early infection group, both for IgG (*p* = 0.0111 and *p* = 0.0072, respectively) and IgM class (*p* = 0.0072 and *p* = 0.0019, respectively; Fig. 2).

It should be also noted that in each group, we found a patient with a double aPL positivity.

**Fig. 3:**
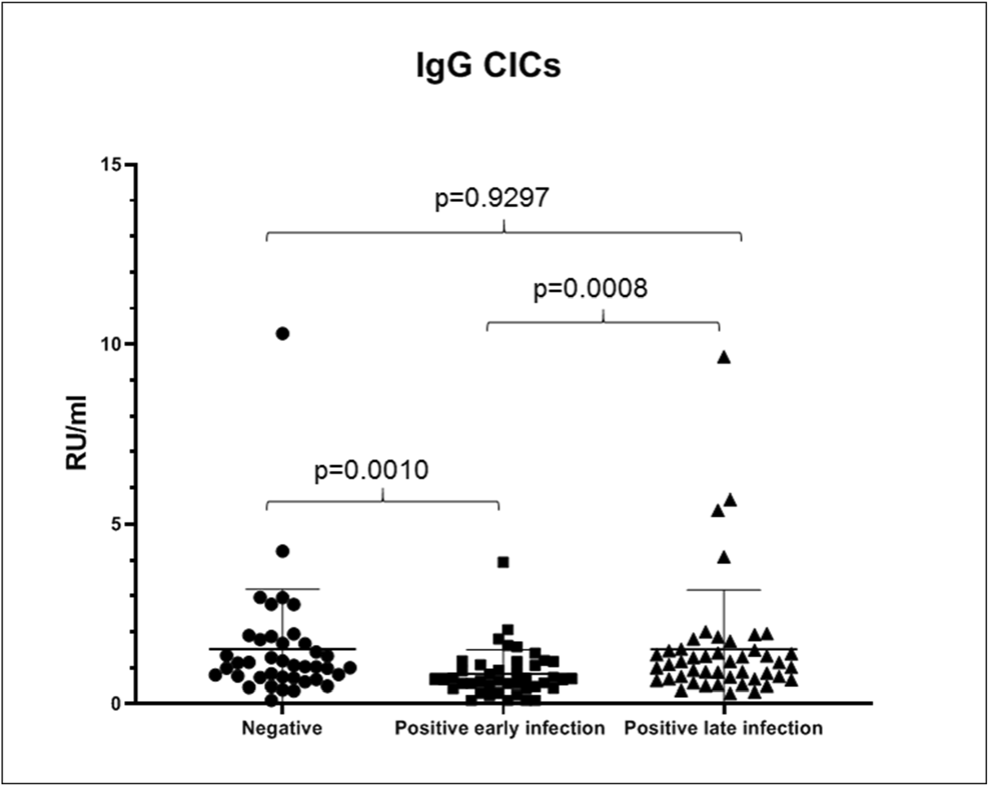
IgG circulating immune complex (CIC) results in control group, positive early infection group, and positive late infection group (Kruskal-Wallis *p* value = 0.0008)

At last, IgG CIC ELISA immunoassay was performed, and results are shown in Fig. 3. Also in this case, a statistical significance among the groups has been found (*p* = 0.0008). Median concentrations are similar between negative and late infection groups (1.045 RU/ml; range 0.1000–10.30 RU/ml, and 1.120 RU/ml; range 0.2900–9.660 RU/ml, respectively), whereas early infection group has a lower median concentration (0.66 RU/ml; range 0.1000–3.940 RU/ml) (Table 1), leading to significant *p* values when it was compared with negative control group and late infection group (*p* = 0.001 and *p* = 0.0008, respectively). No IgG CIC positivity was found in any patient with the manufacturer’s recommended cut-off value (> 20 RU/ml).

## Discussion

SARS-CoV-2 may predispose patients to pro-inflammatory and hypercoagulable states and increased risk of thrombotic events and coagulation abnormalities named “COVID-19 Associated Coagulopathy”. Pathophysiological mechanisms of CAC remain uncertain and are under intensive investigation [2–6].

Preliminary evidences of a possible correlation between anti-phospholipid antibodies and coagulopathy in COVID-19 patients derived from Zhang et al., who reported three cases with thrombosis, aCL, and aβ2GP1 positivity only for IgA class [13]. It should be noticed that lack of IgG and/or IgM aCL and aβ2GP1 precludes the possible role of anti-phospholipid antibodies in CAC, and furthermore, the analyzed patients had a history of cardiovascular disease episodes, which increased itself the risk of subsequent thrombosis events.

Based on these observations, Harzallah et al. tested IgG/IgM aCL, IgG/IgM aβ2GP1, and LAC; LAC positivity was found in 45% of patients, whereas IgG and/or IgM aCL and aβ2GP1 were detected in only 10% [14]. Some authors, however, highlight that high levels of CRP interfere with LAC detection methods [15]. Nevertheless, other studies confirmed a low aPL prevalence, suggesting that they might not be involved in COVID-19 coagulopathy and thrombosis mechanism [16, 17].

Notwithstanding, combination of the various aPL criteria and antibody profiles could be useful to better characterize the risk assessment of thrombotic events in COVID-19 patients [18].

In our data, we have first identified few positive IgG/IgM aCL and IgG/IgM aβ2GP1 patients in all groups, according to manufacturer’s recommended cut-off value (> 20 CU).

As previous studies have shown that moderate to high titers of aPLs display better clinical significance [7], we then reanalyzed data with a more stringent cut-off value (> 40 CU): aPL positivity was confirmed only in two negative controls. Therefore, lack of uniformity and the low percentage of positive cases in IgG/IgM aCL and IgG/IgM aβ2GP1 assays preclude a possible role of aCL and aβ2GP1 antibodies in our cohort.

Likewise, IgG/IgM anti-prothrombin and IgG/IgM anti-annexin-V immunoassays showed few positive cases. Interestingly, IgG/IgM anti-prothrombin and IgG/IgM anti-annexin-V data show that distribution of positive case number increases in late infection patients, significantly in anti-annexin-V results, suggesting a possible role for these anti-phospholipid antibodies in disease course. In fact, it has been reported that aPLs can arise transiently in some patients with critical illness and SARS-CoV-2 infection (disappearing in a few weeks) [19]; as well as in other genetically predisposed patients, they could trigger a “COVID-19-induced-APS-like-syndrome” or other autoimmune diseases [20, 21]. In addition, a recent review proposed follow-up studies on patients recovered from COVID-19, to identify a possible late occurrence of secondary APS in the course of the disease or during the recovery, which could increase thrombotic risk, especially for older patients [22].

Unfortunately, we could not perform a longer-term follow-up.

To note, LAC positivity was found only in two late infection group patients (one with IgM anti-prothrombin positivity and one with IgM anti-annexin-V positivity).

Patients with any aPL positivity were not admitted to ICU, suggesting that presence of these antibodies is not associated to disease severity.

Regarding IgG CIC immunoassay, we have identified no positive cases in all patients’ groups and negative control group. Since CICs are involved in inflammatory phenomena, we would have expected a significant increase, especially in positive early infection group. Conversely, IgG CIC median concentration compared with negative control group decreased from 1.045 to 0.66 RU/ml. Notably, in positive late infection group, IgG CIC median concentration increased to that of negative control group (1.120 RU/ml). These data could be explained with a possible CIC tissue precipitation in inflammation early phases and a subsequent restoring of the normal CIC concentration, in accordance to patients’ clinical manifestation improvement. Nevertheless, our results suggest that in COVID-19, IgG CICs could not be considered as possible infection markers.

SARS-CoV-2 pandemic requires the identification of reliable and significant markers to quickly discriminate COVID-19 patients with general worsening of clinical conditions and increased risk of developing thrombotic events and coagulopathy abnormalities. Unfortunately, our results showing a low anti-phospholipid antibody prevalence pointed out that aPLs could not be considered as valid disease markers, considering that a higher clinically significant cut-off value did not identify any positivity in the infection groups.

Regrettably, our work has several limitations. The study population has been chosen on the basis of available samples previously detected for anti-SARS-CoV-2 serological assays; therefore, it includes healthy subjects as a control group. It is well known that aPL positivity is often associated to infectious diseases; thus, a more informative comparison could have been between COVID-19 patients and those affected by other pneumological diseases. According to hospital data access policy, we cannot provide any further information on medical records and on the clinical status of the patients, except for those results regarding laboratory medicine department.

In conclusion, our data show a low aPL prevalence in accordance with previous studies, suggesting that these autoantibodies might not be involved in the pathogenesis of CAC, but they could arise transiently in COVID-19 patients.

These data could have a potential clinical implication in SARS-CoV-2 infection, proposing that even though autoantibodies are transient, they may still have a thrombotic potential in genetically predisposed COVID-19 patients. Long-term follow-up and prospective evaluations of those aPLs should be performed to verify their persistence and pathogenicity.

## Data Availability

Not applicable.
